# Diagnosis of Eosinophilic Esophagitis at the Time of Esophageal Food Impaction

**DOI:** 10.3390/jcm12113768

**Published:** 2023-05-31

**Authors:** Christina Lee, Tyson J. Sievers, Byron P. Vaughn

**Affiliations:** 1 Department of Medicine, University of Minnesota, 420 Delaware St. SE, MMC 36, Minneapolis, MN 55455, USA; 2 Division of Gastroenterology, Hepatology, and Nutrition, University of Minnesota, 420 Delaware St. SE, MMC 36, Minneapolis, MN 55455, USA

**Keywords:** upper endoscopy, esophageal diseases, esophagitis, food bolus

## Abstract

Background: Esophageal food impactions (EFI) often precede a diagnosis of eosinophilic esophagitis (EOE). Current guidelines suggest obtaining esophageal biopsies upon suspicion of EOE, treating with proton pump inhibitor (PPI), and repeating esophagogastroduodenoscopy (EGD). This study was conducted to determine provider practice patterns with these mentioned recommendations at the time of EFI. Methods: In this retrospective study, key outcomes were the proportion of patients who had EOE mucosal biopsies, EOE diagnosis, PPI initiation, and recommendations and completions of repeat EGD. Differences in outcomes among age, sex, race, off-hours time of procedure, and trainee involvement were examined. EOE diagnosis predictors were explored with logistic regression. Results: Twenty-nine percent of the patients had esophageal biopsies taken at the time of index EGD (iEGD). Sixteen patients were diagnosed with EOE at the time of index EFI, while fourteen patients were diagnosed on subsequent EGDs. Among those diagnosed with EOE at iEGD, 94% were placed on PPI. Of patients with confirmed EOE on index biopsy, 63% of patients were recommended repeat EGD, of which 50% completed it within 90 days. Older age was protective of EOE diagnosis while no GERD history and endoscopist suspicion of EOE predicted diagnosis of EOE. Conclusions: Endoscopists uncommonly take biopsies at the time of EFI, which may delay diagnosis and treatment of EOE.

## 1. Introduction

Esophageal food impactions (EFI) are the presenting manifestation of eosinophilic esophagitis (EOE) in up to 50% of EFI patients [1,2,3]. While EFI frequently requires esophagogastroduodenoscopy (EGD) to relieve the obstruction, the diagnosis of EOE can be delayed [4].

Despite the relationship between EOE and EFI [5,6], there is no standard diagnostic and therapeutic practice for EOE at the time of EFI. The 2018 AGREE Conference and 2020 AGA guidelines provide flexible recommendations that suggest obtaining esophageal mucosal biopsies from two or more esophageal levels upon suspicion of EOE, such as in the setting of an EFI [7]. These guidelines suggest treatment with a proton pump inhibitor (PPI) upon diagnosis of EOE, followed by repeat endoscopy (EGD) to assess for treatment response [8].

Despite recommendations for obtaining esophageal mucosal biopsies upon clinical suspicion for EOE, clinicians may be reluctant to biopsy in the setting of EFI. A survey of US-based gastroenterologists identified 34% of gastroenterologists obtaining biopsies at the time of EFI, and of those obtaining biopsies, 46% of gastroenterologists waited for histology results before starting PPI [9]. The inconsistency in obtaining esophageal mucosal biopsies can delay EOE diagnosis and treatment and lead to unnecessary repeat EGDs.

The aim of this study is to quantify gastroenterologists’ practice patterns for obtaining biopsies and treating EOE, recommending follow-up EGDs for EOE at the time of EFI, and to investigate how they impact diagnosis and treatment of EOE, as well as identify predictors for EOE diagnosis.

## 2. Materials and Methods

### 2.1. Patient Population

We performed a retrospective study at the University of Minnesota Medical Center. This study was approved by the University of Minnesota Institutional Review Board. This retrospective chart review study involving human participants was in accordance with the ethical standards of the institutional and national research committee and with the 1995 Helsinki Declaration and its later amendments or comparable ethical standards. Due to the nature of this retrospective study and the preserved anonymity of the patients, a waiver of informed consent was obtained from and approved by the University of Minnesota Institutional Review Board.

The database included patients presenting to the University of Minnesota Medical Center, aged 18 years and older, with an endoscopically confirmed EFI between 2009 and 2020. Patients were excluded if no EFI or non-food impactions were present on index EGD (iEGD), if medical records were incomplete, or if patients had a history of esophageal carcinoma.

### 2.2. Outcome Measures

The goals of this study were to identify the proportion of patients who had EOE mucosal biopsies, EOE diagnosis, PPI initiation, recommendations and completions of repeat EGD. Demographic information, EGD characteristics, and outcomes were manually extracted from the electronic medical record. Provider suspicion for EOE was extracted from the procedure report. Provider suspicion included documentation of endoscopic findings of EOE (e.g., concentric rings, linear furrows) along with a provider interpretation stating the findings were “suspicious for” or “consistent with” EOE. Diagnosis of EOE was based on the provider’s overall impression of EOE and documented 15 or more eos/hpf in at least one microscopy field [7]. The PPI prescription was gathered from the discharge summaries, orders, and EGD reports. The initial EGD with an esophageal food impaction was the iEGD, and the subsequent EGD was the repeat EGD. The recommendation for repeat EGD was gathered from iEGD reports, discharge summaries, and gastroenterology consult notes.

### 2.3. Statistical Analysis

Baseline characteristics were summarized as proportions or mean/median and standard deviation/interquartile range based on normalcy of the data. Percent totals of EFI patients were calculated for obtained biopsies, EOE diagnosis, prescribed PPI, recommended and obtained follow-up EGDs. Through a univariate analysis, patient demographics including age, sex, race (white vs. non-white), off-hours EGD (i.e., not from Monday to Friday from 8:00 a.m–5:00 p.m), and trainee involvement were explored along with iEGD characteristics, such as biopsy, EOE diagnosis, PPI, and repeat EGD. Age was explored continuously and with relevant cut-offs identified by receiver operating curve. If age was associated with an outcome at a *p* < 0.1, then an ROC curve was obtained and Youden’s J statistic was used to find the value corresponding to maximum specificity and sensitivity. EOE diagnosis at any time after the iEGD was the primary outcome. Exploratory logistic regression was performed to identify predictors of EOE diagnosis at the time of iEGD. The following predictors of EOE diagnosis were analyzed a priori: age, sex, BMI, race, trainee involvement, no GERD history, off-hours EGD procedure time, PPI use prior to iEGD, endoscopic suspicion of EOE at iEGD, endoscopic ring or stricture at iEGD, endoscopic esophagitis at iEGD. Parameters with a *p*-value of <0.1 were included in a multivariate analysis. In addition, age and sex were included a priori into the multivariate analysis. Parameters with a *p*-value of <0.05 were considered significant. Statistical analysis was performed using JMP Pro 15.

## 3. Results

**Figure 1:**
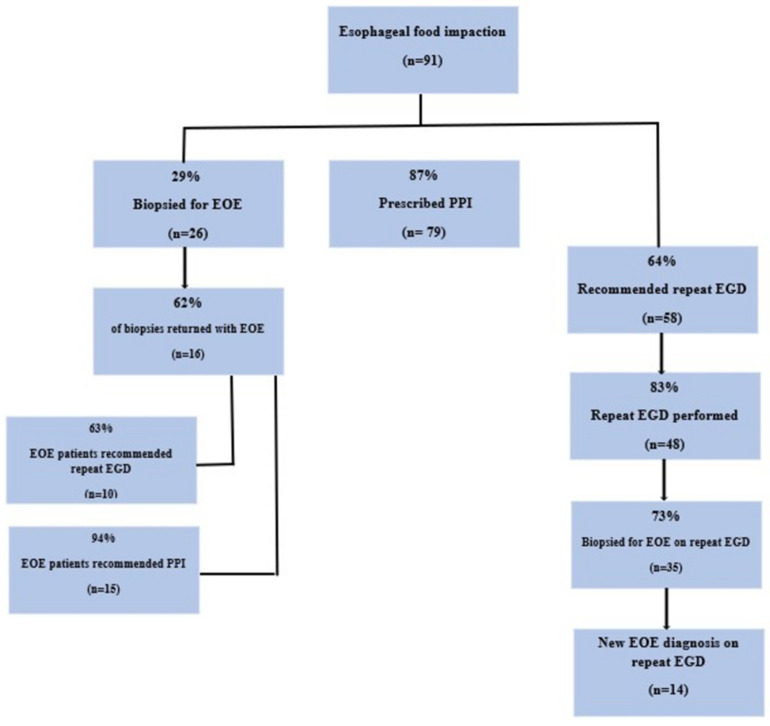
Visual representation of biopsies, prescribed PPI, recommended and repeat EGD in esophagel food impaction patients. Legend: Graphical representation of obtained biopsies, prescribed PPI, and recommended and performed repeat EGD amongst the total of 91 esophageal food impaction patients. Abbreviation: EOE: eosinophilic esophagitis, PPI: proton pump inhibitor, n: total number of patients.

A total of 223 patients were identified from the University of Minnesota database with food impaction cases between the years 2009–2020. Three patients were excluded due to a history of esophageal carcinoma. Additionally, 129 patients were excluded for presenting with either non-food impactions, no confirmed EFI, and/or incomplete medical records. Ninety-one patients met the inclusion criteria for our cohort. Baseline characteristics are presented in Table 1. The main breakdown of biopsies, prescribed PPI, and recommended and obtained repeat EGD are presented in Figure 1.

Only 29% (26/91) of patients had esophageal biopsies taken at the time of iEGD, despite 41% (37/91) having an endoscopic suspicion of EOE. Among patients with an endoscopic suspicion for EOE, biopsies were performed at iEGD only 20% (18/37) of the time. There were no significant differences in sex, race, off-hours EGD, or trainee involvement in those with or without biopsies. Age was borderline associated with taking biopsies (*p* = 0.06). ROC analysis identified an age of 40 as a relevant cut off. Younger patients (≤40) were more likely to have biopsies (*p* = 0.04). In total, 32% (11/34) of biopsies were taken from the GI endoscopy suite, 57% (8/14) were obtained from the ED, and 15% (6/40) were obtained from the operation room (OR). Compared to non-OR locations, biopsies were significantly less likely to be taken in the OR (*p* = 0.01).

Of the 26 patients with esophageal biopsies on iEGD, 62% (16/26) were diagnosed with EOE on iEGD. Of the 65 patients who did not have a biopsy at iEGD, 37% (24/65) had a subsequent EGD, yielding an additional 14 diagnoses of EOE. Overall, 33% (30/91) were diagnosed with EOE at some point in the cohort. Overall, there were 41 individuals who never had esophageal biopsies, and a precise etiology for EFI was not identified. There were no statistical differences in sex, race, off-hours EGD, or trainee involvement in those with and without EOE diagnosis. Age was significantly associated with EOE diagnosis; the median age at iEGD of those with EOE was 39 (IQR: 30, 46) vs. 59 (IQR: 43, 72) for those without (*p* < 0.001). ROC analysis identified the age of 51 or younger as predictive of EOE (OR: 14 95% CI: 3.4, 50, *p* < 0.0001).

Most patients, 87% (79/91), were discharged on PPI therapy after the iEGD with prescribed doses ranging from 20 mg to 80 mg in 24 h, the latter being the most frequently prescribed dose (43%, 39/91). Of those with a suspicion of EOE documented in the procedure report at the time of iEGD, 92% (34/37) were discharged on PPI therapy vs. 85% (46/54) without suspicion of EOE (*p* = 0.5). Among those diagnosed with EOE at iEGD, 94% (15/16) were placed on PPI therapy before the pathology results returned. Men were more likely to be placed on PPI vs. women (60% vs. 40%, *p* = 0.05). Otherwise, there were no statistical differences with age, race, off-hours EGD, or trainee involvement in those with or without PPI prescriptions at discharge.

Following iEGD, 64% (58/91) of patients had documentation in the chart to receive a repeat EGD. In total, 55% (32/58) underwent a repeat EGD within 90 days of iEGD while 53% (48/91) had a repeat EGD at some point in follow-up. Of the repeat EGD patients, 65% (31/48) were scheduled follow-ups from prior EFI, 21% (10/48) were scheduled for dysphasia, and 15% (7/48) were scheduled for recurrent EFI. However, only two of the subsequent EFIs were ultimately diagnosed with EOE, one of which was not a new diagnosis. Of the 16 patients confirmed with EOE on initial biopsy, 63% (10/16) patients were recommended repeat EGD, and 50% (5/10) completed a repeat EGD within 90 days. There were no differences in age, sex, race, off-hours EGD, or trainee involvement with documentation for recommendation of repeat EGD; however, none (0/4) of the non-white patients (two Somalian, one Native American, one Asian) who were recommended repeat EGD in the cohort had a repeat EGD within 90 days (*p* = 0.03).

Ultimately, at the time of iEGD, only 20% (18/91) of patients had the combination of esophageal biopsies, PPI therapy recommendation, and repeat EGD recommendation.

### Predictors of EOE Diagnosis

Univariate and multivariate analyses of exploratory predictors for EOE diagnosis are presented in Table 2. Older age was protective of EOE diagnosis, while the lack of a GERD history and endoscopist suspicion of EOE predicted diagnosis of EOE.

## 4. Discussion

EOE is one of the leading causes of recurrent EFI [1,2,3]. The EGD at the time of food impaction is an important opportunity to diagnose, treat, and recommend follow-up to determine the underlying cause of EFI as EOE.

Our results suggest that one third of patients with EFI have underlying EOE. While it is encouraging that most patients are discharged on a PPI, we identified several care gaps that resulted in diagnostic delays of EOE. Only a third of patients underwent biopsies at the index EGD and only two thirds of patients were recommended to have a repeat EGD following EFI. Of concern, around half of the patients who were recommended repeat EGD received a repeat EGD within 90 days of EFI. These missed opportunities resulted in delayed diagnosis and unnecessary repeat EGDs, and overall poor patient care. Half of the patients with EOE in our study were not diagnosed until the repeat EGD.

Our findings are in line with the prior literature suggesting that approximately only one third of gastroenterologists take esophageal biopsies at the time of EFI [9]. We hypothesize that there are two main reasons for this to occur. First, there may be a perception that biopsies taken at the time of EFI could increase complications. Most complications are rare, including esophageal mucosal tears, intubation for airway protection, and procedural agitation [10]. Frank perforations are uncommon and are not linked to biopsies. Biopsies from non-inflamed esophageal mucosa should have the same risk as a non-EFI setting and still can provide diagnostic support for EOE. Provider discretion is still needed as some prolonged EFIs may not be appropriate to biopsy. We suspect the other reason for lack of biopsies falls into “provider practice patterns” and “therapeutic inertia”. These include factors such as provider knowledge to take biopsies and the location of the procedure. Biopsies are significantly less likely to be taken in the OR setting. It may be that sicker patients have an EGD for EFI in the OR, and thus providers are more concerned for complications. While provider discretion is always important, our data suggest that younger patients with lack of GERD and/or any suspicion of EOE endoscopically should be biopsied at the time of EFI. We recommend, at the time of EFI, to take biopsies from two or more levels of non-inflamed esophageal mucosa, consistent with the AGA guidelines.

Prior work suggests that the rate of those lost to follow-up is greater when biopsies are not taken [11]. In general, repeat EGD occurs around 50% of the time, similar to our study [11]. Chang et al. identified that without biopsies, around 80% do not follow-up [11], although our study found that 48% of patients without biopsies did not have repeat EGD. In our cohort, non-white race was associated with a lack of repeat EGD. This is generally consistent with compliance to endoscopic procedures in general [12]. Due to small numbers, we were unable to assess for relevant confounding on this association nor able to perform statistical methods to minimize for possible confounders of race. There are a number of systematic issues related to endoscopy compliance and race [13]. Given historical trust issues and barriers to care (insurance, cost, transportation), special attention should be given to ensuring aide with compliance in follow-up recommendations for non-white patients.

Our study also investigated for predictors of EOE diagnosis. Gastroenterologists’ suspicion of EOE appears to predict diagnosis of EOE. In addition, the rate of biopsies increases with the suspicion of underlying EOE [9]. However, EOE commonly has no endoscopic findings. Thus, the presence of EFI alone should raise suspicion for EOE diagnosis. Additionally, risk factors for EOE identified by us were similar to those previously published. Younger age and no prior history of GERD suggest EOE [1]. As EOE can lead to strictures in the esophagus, the presence of a stricture should not dissuade a gastroenterologist from considering EOE. In sum, given the diagnostic yield of obtaining biopsies at the time of EFI, we advocate for biopsies at the time of EFI from two esophageal levels.

Our study has several important limitations. EOE is seen in a wide spectrum of ages, including children, adolescents, and adults [14]. The cohort of patients in our study, comprised mostly of middle-aged, white individuals, may not be generalizable to other communities. Our study is also limited by the inherent confounding of retrospective observational studies. Certain disease mediators or confounders, such as food allergies, were not available for review in the electronic medical records; likewise, it is possible there was diagnostic misclassification with EOE and other diseases causing esophageal eosinophilia. It is also possible that EOE diagnoses were missed due to the lack of clinical follow-up for individuals who were never biopsied. Additionally, due to small numbers, we could not control for important covariates such as race. While documentation of provider suspicion of EOE did predict EOE diagnosis, we did not have reliable data on the specific endoscopic findings that were most predictive. Our findings should be considered exploratory, and thus we recommend further examination of larger national, prospective cohorts.

In summary, we found that it is uncommon for gastroenterologists to obtain biopsies at the time of an EFI. While many patients are discharged on PPI and recommended follow-up EGD, care gaps still exist, which can likely be addressed with further education and guideline development. Although research on EOE has expanded dramatically over the past 20 years, there still remain important care gaps. We suggest implementation of guidelines to be a focus for future research endeavors. A high percentage of EFI patients will have a diagnosis of EOE. The diagnosis of EOE can be expedited if biopsies are obtained at the initial presentation. These data highlight the need for increased provider educations and standardized management and recommendations after EFI. Future studies should focus on identifying the optimal means to reduce these care gaps.

## Figures and Tables

**Table 1:** Baseline characteristics of esophageal food impaction patients.

Characteristic	N (%)
Male	51/91 (56.04%)
Mean age (SD) at index EGD	50.30 (18.68)
Mean BMI (SD)	29.16 (7.35)
Race	
Caucasian	84/91 (92.31%)
Non-Caucasian	7/91 (7.70%)
Proton pump inhibitor use prior to index EGD	19/91 (20.88%)
History of cancer	3/91 (3.30%)
Metastatic prostate cancer	1/3 (33.33%)
Pancreatic cancer	1/3 (33.33%)
Lung cancer	1/3 (33.33%)
History of GERD	27/91 (29.67%)
History of food impaction	7/91 (7.70%)
Prior endoscopy	27/91 (29.67%)

Legend: The percentages of esophageal food impaction patients with each baseline characteristics are presented above. Notably, this study was comprised mainly of overweight white male patients with mean age of index EGD at 50 years old.

**Table 2:** Parameters and index EGD characteristics of esophageal food impaction patients.

Parameter	Univariate *p*-Value	Multivariate *p*-Value	Adjusted OR (CI)
Age	0.008 *	0.003 *	0.94 (0.90, 0.98)
Sex	0.8		
BMI	0.9		
Race	0.7		
Trainee involvement	0.7		
No GERD history	0.01 *	0.04 *	4.7 (1.1, 20.6)
Off-hours EGD	0.04 *	0.08	3.6 (0.84, 15.6)
PPI use prior to index EGD	0.4		
Endoscopic suspicion of EOE at index EGD	<0.0001 *	0.05 *	3.2 (1.02, 10.1)
Endoscopic ring or stricture at index EGD	0.8		
Endoscopic esophagitis at index EGD	0.9		

Legend: The univariate and multivariate *p*-values for the patients and index EGD characteristics are presented above, followed by adjusted OR values. Age, no GERD history, and endoscopic suspicion of EOE at index EGD were all statistically significant (*p* < 0.05) * with corresponding confidence intervals not including the value 1. Off-hours EGD (performed on nights and weekends) was only statistically significant with the univariate analysis.
